# Supplementary material for: Transcriptomic Analysis of Osmotic Stress-Tolerant Somatic Embryos of Coffea arabica L. Mediated by the Coffee Antisense Trehalase Gene: A Marker-Free Approach
Source: Int J Mol Sci. 2025 Sep 21;26(18):9224. doi: 10.3390/ijms26189224 (PMC12471068; doi:10.3390/ijms26189224)
Supplement: Supplementary file 1 [file ijms-26-09224-s001.zip › Supplementary Table S1 KEEGs.pdf]

Article

Supplementary Table S1

Transcriptomic analysis of osmotic stress tolerant somatic embryos of *Coffea arabica* L. mediated by the coffee antisense *Trehalase* gene: A Marker free approach.

Eliana Valencia-Lozano[1]*, Aarón Barraza[2], Jorge Ibarra[3], John P. Délano-Frier[3], Norma Martínez-Gallardo[3], Anali Gamez-Escobedo[4] and José Luis Cabrera-Ponce[5]*

1. Supplementary table S1. KEEGs

No	KEEG Pathway	observed gene count	Matching proteins in your network (labels)
1	Biosynthesis of secondary metabolites	56	A0A068TL75,A0A068TM02,A0A068TNZ2,A0A068TPC0,A0A068TPJ2,A0A068TPN7,A0A068TQ20,A0A068TQG5,A0A068TRJ4,A0A068TS59,A0A068TSC2,A0A068TXJ5,A0A068TXQ8,A0A068TXS1,A0A068TYM2,A0A068TYT9,A0A068TZ70,A0A068TZQ1,A0A068U2A4,A0A068U2G9,A0A068U2N3,A0A068U357,A0A068U3V8,A0A068U6K1,A0A068U8A0,A0A068U944,A0A068U9D0,A0A068U9V1,A0A068UD94,A0A068UDD0,A0A068UDZ3,A0A068UET6,A0A068UH55,A0A068UIW2,A0A068ULH0,A0A068UR82,A0A068UTX2,A0A068UW10,A0A068UZB5,A0A068V426,A0A068V5Q9,A0A068V643,A0A068V7Y1,A0A068V9Q2,A0A068VBS4,A0A068VDX2,A0A068VEF1,A0A068VEU9,A0A068VFV1,A0A068VH53,A0A068VH54,A0A068VL45,A0A068VLP2,A0A068VLV0,A0A068VM15,CCoAOMT1
2	DNA replication	15	A0A068TN78,A0A068TNS0,A0A068TTG1,A0A068U1V9,A0A068U916,A0A068UGH6,A0A068UNF1,A0A068UV29,A0A068UXQ0,A0A068V150,A0A068V2B3,A0A068V4C9,A0A068VDS9,A0A068VHF0,A0A068VIW0
3	Homologous recombination	13	A0A068TLA1,A0A068TQC9,A0A068U3I1,A0A068U440,A0A068U850,A0A068UCZ6,A0A068UXQ4,A0A068V2B3,A0A068V4C9,A0A068V703,A0A068VBY6,A0A068VHF0,A0A068VKU9

4	Metabolic pathways	77	A0A068TL75,A0A068TM02,A0A068TNZ2,A0A068TPC0,A0A068TPJ2,A0A068TPN7,A0A068TQ20,A0A068TQG5,A0A068TRA1,A0A068TRJ4,A0A068TS59,A0A068TSC2,A0A068TU74,A0A068TXJ5,A0A068TXS1,A0A068TYM2,A0A068TYT9,A0A068TZ70,A0A068TZQ1,A0A068TZW2,A0A068U1E4,A0A068U1M9,A0A068U1N4,A0A068U2A4,A0A068U2G9,A0A068U2S6,A0A068U338,A0A068U357,A0A068U3V8,A0A068U6K1,A0A068U8A0,A0A068U944,A0A068U9V1,A0A068UD94,A0A068UDD0,A0A068UDZ3,A0A068UEJ9,A0A068UET6,A0A068UG29,A0A068UGH3,A0A068UH55,A0A068UIN3,A0A068UIW2,A0A068UJ88,A0A068ULH0,A0A068UM62,A0A068UMU7,A0A068UQ78,A0A068UR82,A0A068UTX2,A0A068UW10,A0A068UW34,A0A068UY65,A0A068UZB5,A0A068UZW0,A0A068V0Y1,A0A068V3M2,A0A068V426,A0A068V5Q9,A0A068V5V2,A0A068V643,A0A068V7Y1,A0A068V7Y8,A0A068V961,A0A068VBS4,A0A068VCH1,A0A068VDT3,A0A068VDX2,A0A068VEF1,A0A068VEU9,A0A068VFV1,A0A068VH53,A0A068VL45,A0A068VLP2,A0A068VLV0,A0A068VM15,CCoAOMT1
5	Glycerolipid metabolism	10	A0A068TL75,A0A068TXQ8,A0A068U2N3,A0A068UDD0,A0A068UDZ3,A0A068UIW2,A0A068UJ88,A0A068UQ78,A0A068V426,A0A068VLP2
6	Phenylpropanoid biosynthesis	15	A0A068TPC0,A0A068TPN7,A0A068TQ20,A0A068TQG5,A0A068TSC2,A0A068TZ70,A0A068U3D1,A0A068UET6,A0A068UTX2,A0A068VBS4,A0A068VEU9,A0A068VFV1,A0A068VH54,A0A068VM15,CCoAOMT1
7	Mismatch repair	7	A0A068TQC7,A0A068TR00,A0A068UNF1,A0A068V235,A0A068V2B3,A0A068V4C9,A0A068VHF0
8	Glycerophospholipid metabolism	8	A0A068TL75,A0A068TXQ8,A0A068U2N3,A0A068UDD0,A0A068UDZ3,A0A068UIW2,A0A068V426,A0A068VLP2
9	Ubiquinone and other terpenoid-quinone biosynthesis	5	A0A068TPN7,A0A068UET6,A0A068UTX2,A0A068V5Q9,A0A068VFV1
10	Indole alkaloid biosynthesis	2	A0A068VL45,AAS
11	Glucosinolate biosynthesis	3	A0A068U9D0,A0A068U2A4,A0A068VDX2

12	Phenylalanine metabolism	3	AAS,TAT3,A0A068VM15
13	Glycolysis / Gluconeogenesis	5	ENOC,LOS2,PKP-ALPHA,HCEF1
14	Arginine biosynthesis	3	WIN1,ARGAH1,GDH1
15	Biosynthesis of amino acids	12	A0A068TM02,A0A068TNZ2,A0A068TPJ2,A0A068TS59,A0A068TXS1,A0A068TYM2,A0A068U2A4,A0A068U8A0,A0A068UH55,A0A068ULH0,A0A068V643,A0A068V7Y1
16	Flavonoid biosynthesis	5	A0A068TQG5,A0A068VEU9,A0A068VFV1,A0A068VH53,CCoAOMT1
17	Stilbenoid, diarylheptanoid and gingerol biosynthesis	4	A0A068TQG5,A0A068VEU9,A0A068VFV1,CCoAOMT1
18	Galactose metabolism	5	A0A068U1M9,A0A068UW10,A0A068UY65,A0A068V3M2,A0A068V5V2
19	Starch and sucrose metabolism	8	A0A068TXJ5,A0A068TYT9,A0A068TZQ1,A0A068U357,A0A068U3V8,A0A068U944,A0A068U9V1,A0A068UW10
20	Valine, leucine, and isoleucine biosynthesis	3	A0A068U2A4,A0A068U8A0,A0A068V7Y1
21	2-Oxocarboxylic acid metabolism	5	A0A068TPJ2,A0A068U2A4,A0A068U8A0,A0A068V7Y1,A0A068VDX2
22	Nucleotide excision repair	7	A0A068TTG1,A0A068ULV6,A0A068UNF1,A0A068V1A7,A0A068V2B3,A0A068V4C9,A0A068VHF0
