# Supplementary material for: Transcriptomic Analysis of Osmotic Stress-Tolerant Somatic Embryos of Coffea arabica L. Mediated by the Coffee Antisense Trehalase Gene: A Marker-Free Approach
Source: Int J Mol Sci. 2025 Sep 21;26(18):9224. doi: 10.3390/ijms26189224 (PMC12471068; doi:10.3390/ijms26189224)
Supplement: Supplementary file 1 [file ijms-26-09224-s001.zip › Supplementary Table S12 Lethal -plants.pdf]

Article

Supplementary Table S12

Transcriptomic analysis of osmotic stress tolerant somatic embryos of *Coffea arabica* L. mediated by the coffee antisense *Trehalase* gene: A Marker free approach.

Eliana Valencia-Lozano[1]*, Aarón Barraza[2], Jorge Ibarra[3], John P. Délano-Frier[3], Norma Martínez-Gallardo[3], Anali Gamez-Escobedo[4] and José Luis Cabrera-Ponce[5]*

1. Supplementary Table S12. Embryo lethal genes

No	**ID *A. thaliana***	**ID *Coffea arabica***	**Associated function**	**Modules**
1	*CDC48A*	*A0A068UGL5*	Cell division control protein 48 homolog A	Somatic Embryo
2	*NFYA9*	*A0A068VE79*	Nuclear transcription factor Y subunit A-9	Somatic Embryo
3	*NFYA8*	*A0A068VIL0*	Nuclear transcription factor Y subunit A-8	Somatic Embryo
4	*ABI3*	*A0A068U8A0*	B3 domain-containing transcription factor ABI3	Somatic Embryo
5	*FUS3*	*A0A068V7Y1*	B3 domain-containing transcription factor FUS3	Somatic Embryo
6	*WOX2*	*A0A068UL49*	WUSCHEL-related homeobox 2	Somatic Embryo
7	*UBP14*	*A0A068UBB6*	Ubiquitin carboxyl-terminal hydrolase 14	Somatic Embryo
8	*MCM2*	*A0A068VDS9*	DNA replication licensing factor MCM2	Cell Cycle
9	*MCM3*	*A0A068VIW0*	DNA replication licensing factor MCM3	Cell Cycle
10	*MCM4*	*A0A068TNS0*	DNA replication licensing factor MCM4	Cell Cycle
11	*MCM5*	*A0A068V150*	DNA replication licensing factor MCM5	Cell Cycle
12	*MCM6*	*A0A068U916*	DNA replication licensing factor MCM6	Cell Cycle
13	*MCM7*	*MCM7-2*	DNA replication licensing factor MCM7	Cell Cycle
14	*POLA*	*A0A068U1V9*	DNA polymerase alpha catalytic subunit	Cell Cycle
15	*POLA2*	*A0A068UXQ0*	DNA polymerase alpha subunit B	Cell Cycle
16	*DPB2*	*A0A068TTG1*	DNA polymerase epsilon subunit B	Cell Cycle
17	*CDT1A*	*A0A068U5J0*	CDT1-like protein a, chloroplastic	Cell Cycle
18	*T10F20.10*	*A0A068TR00*	5'-3' exonuclease family protein.	Cell Cycle
19	*MAP65-3*	*A0A068U9S3*	65-kDa microtubule-associated protein 3	Cell Cycle
20	*ORC5*	*A0A068UMG4*	Origin of replication complex subunit 5	Cell Cycle
21	*MUS81*	*A0A068V703*	Crossover junction endonuclease MUS81	Cell Cycle
22	*EMB2656*	*A0A068U0F3*	ARM repeat superfamily protein.	Cell Cycle
23	*SMC2-1*	*A0A068V0V7*	Structural maintenance of chromosomes protein 2-1	Cell Cycle
24	*ESP1*	*A0A068TNC3*	Separase	Cell Cycle
25	*AGO1*	*A0A068TRH0*	Protein argonaute 1	Chromatin Remodeling

26	*EMB2271*	*A0A068UBQ4*	U3 snoRNP-associated protein-like EMB2271	Chromatin Remodeling
27	*ENO2*	*A0A068V643*	Bifunctional enolase 2/transcriptional activator	Carbon Metabolism
28	*PGDH1*	*A0A068TM02*	D-3-phosphoglycerate dehydrogenase 1, chloroplastic	Carbon Metabolism
29	*P5CSB*	*A0A068TXS1*	Delta-1-pyrroline-5-carboxylate synthase B	Glucosinolates, Strictosidine biosynthesis
30	*EMB1674*	*A0A068UQ70*	Protein EMBRYO DEFECTIVE 1674	Generic transcription Pathway
31	*RPL15*	*A0A068TXH8*	50S ribosomal protein L15, chloroplastic	Generic transcription Pathway
