# Supplementary material for: Transcriptomic Analysis of Osmotic Stress-Tolerant Somatic Embryos of Coffea arabica L. Mediated by the Coffee Antisense Trehalase Gene: A Marker-Free Approach
Source: Int J Mol Sci. 2025 Sep 21;26(18):9224. doi: 10.3390/ijms26189224 (PMC12471068; doi:10.3390/ijms26189224)
Supplement: Supplementary file 1 [file ijms-26-09224-s001.zip › Supplementary Table S13 to S23 DOWMREGULATED.pdf]

Article

Supplementary Tables S13 to S23

Transcriptomic analysis of osmotic stress tolerant somatic embryos of *Coffea arabica* L. mediated by the coffee antisense *Trehalase* gene: A Marker free approach.

Eliana Valencia-Lozano[1]*, Aarón Barraza[2], Jorge Ibarra[3], John P. Délano-Frier[3], Norma Martínez-Gallardo[3], Anali Gamez-Escobedo[4] and José Luis Cabrera-Ponce[5]*

1. Supplementary Table S13. Ribosomal Protein

No	*ID A. thaliana*	ID *Coffea arabica*	ID *A. thaliana*
1	*T11J7.11*	A0A068UKB2	Thioesterase/thiol ester dehydrase-isomerase superfamily protein.
2	*CXE17*	A0A068TXD8	Probable carboxylesterase 17
3	*EFL3*	A0A068U3H1	Protein ELF4-LIKE 3
4	*AMSH2*	A0A068UXQ9	AMSH-like ubiquitin thioesterase 2
5	*IVD*	A0A068UFV0	Isovaleryl-CoA dehydrogenase, mitochondrial
6	*TIF3D1*	A0A068TTA2	Eukaryotic translation initiation factor 3 subunit D
7	*Q9LSW0_ARATH*	A0A068U613	dCTP pyrophosphatase 1
8	*Q94KE3_ARATH*	A0A068V3H1	Pyruvate kinase
9	*PFK7*	PFK-5	ATP-dependent 6-phosphofructokinase 7
10	*RIBA1*	A0A068TN07	Bifunctional riboflavin biosynthesis protein RIBA 1, chloroplastic
11	*APY2*	A0A068VER8	Apyrase 2
12	*TBR*	A0A068UPJ1	Protein trichome birefringence
13	*T29E15.11*	A0A068U3W3	NAD(P)-binding Rossmann-fold superfamily protein.
14	*CRL1*	A0A068VF15	NAD(P)-binding Rossmann-fold superfamily protein.
15	*F7K24.190*	A0A068ULV7	NAD(P)-binding Rossmann-fold superfamily protein.
16	*UMK3*	A0A068ULN3	UMP-CMP kinase 3
17	*T11J7.5*	A0A068U3S9	Probable 3-hydroxyisobutyryl-CoA hydrolase 3
18	*3AT2*	A0A068UWA0	#N/D
19	*RTL1*	A0A068US39	RNAse II-like 1.

20	*UGT75C1*	A0A068V3H3	UDP-glycosyltransferase 75C1
21	*RPL5*	A0A068UJR9	50S ribosomal protein L5, chloroplastic
22	*BSD2*	A0A068UPN7	Protein BUNDLE SHEATH DEFECTIVE 2, chloroplastic
23	*F28J7.7*	A0A068TYG3	Mitochondrial ribosomal protein L37.
24	*TCTP1*	A0A068TRC3	Translationally-controlled tumor protein 1
25	*RPL10A*	A0A068U8U1	60S ribosomal protein L10-1
26	*F4JPD8_ARATH*	A0A068UX16	Nucleoside diphosphate kinase.
27	*RPS13B*	A0A068US28	40S ribosomal protein S13-2

2. Supplementary Table S14. Transmembrane transporter activity

No	ID *A. thaliana*	ID *Coffea arabica*	ID *A. thaliana*
28	*KAT3*	A0A068VD00	Potassium channel KAT3
29	*RAP2-11*	A0A068VAH9	Ethylene-responsive transcription factor RAP2-11
30	*POT5*	A0A068V654	Potassium transporter 5
31	*NPF7.3*	A0A068UZA9	Protein NRT1/ PTR FAMILY 7.3
32	*NRT2.4*	A0A068UFU4	High affinity nitrate transporter 2.4
33	*NRT3.1*	A0A068UYM9	High-affinity nitrate transporter 3.1
34	*NPF2.13*	A0A068TVH2	Protein NRT1/ PTR FAMILY 2.13
35	*BAH1*	A0A068VMD7	E3 ubiquitin-protein ligase BAH1
36	*PHT1-4*	A0A068TSN3	Inorganic phosphate transporter 1-4
37	*LPR2*	A0A068TXW8	Multicopper oxidase LPR2
38	*ALS3*	A0A068UDZ9	Protein ALUMINUM SENSITIVE 3
39	*STOP1*	A0A068VI21	Protein SENSITIVE TO PROTON RHIZOTOXICITY 1
40	*ELP*	A0A068UN95	Extensin-like protein.
41	*AZF2*	A0A068TYA8	Zinc finger protein AZF2
42	*CYS2*	A0A068UDJ1	Cysteine proteinase inhibitor
43	*A0A1P8B9B2*	A0A068VKP4	Heavy metal transport/detoxification superfamily protein.
44	*VAB*	A0A068U5A1	VAN3-binding protein.
45	*CHX18*	A0A068UU86	Cation/H(+) antiporter 18
46	*DTX40*	A0A068UTD2	Protein DETOXIFICATION 40.
47	*DTX42*	A0A068VDN0	Protein DETOXIFICATION 42
48	*ZAT6*	A0A068TYA8	Zinc finger protein AZF2
49	*MXC20.12*	A0A068UBB7	AT hook motif-containing protein.
50	*MYB62*	A0A068TWL8	Transcription factor MYB62
51	*WRKY6*	A0A068U7A5	WRKY transcription factor 6

52	*WRKY75*	A0A068UY66	Probable WRKY transcription factor 75
53	*FAD-OXR*	A0A068UVZ0	Berberine bridge enzyme-like 22.
54	*T5J17.9*	A0A068TZP1	Cupredoxin superfamily protein
55	*ACA2-2*	A0A068U4R4	Calcium-transporting ATPase 2, plasma membrane-type
56	*MO2*	A0A068UMB2	Monooxygenase 2.
57	*CER26*	A0A068URP2	Protein ECERIFERUM 26
58	*FAR3*	A0A068UEF7	Fatty acyl-CoA reductase 3
59	*CUT1*	A0A068U3P4	3-ketoacyl-CoA synthase 6
60	*GPAT6*	A0A068UDZ3	Glycerol-3-phosphate 2-O-acyltransferase 6
61	*ABCG11*	A0A068VCQ4	ABC transporter G family member 11
62	*SDI1*	A0A068TT36	Protein SULFUR DEFICIENCY-INDUCED 1
63	*F21C20.170*	A0A068UVZ9	Berberine bridge enzyme-like 18.
64	*KCS4*	A0A068UIQ6	3-ketoacyl-CoA synthase 4.
65	*F12F1.10*	A0A068UA33	Phosphoenolpyruvate carboxylase, putative (DUF506).
66	*T2K10.11*	A0A068TY00	Serine/threonine-protein kinase WNK (With No Lysine)-like
67	*LTP3*	A0A068V2X8	Non-specific lipid-transfer protein 3
68	*KCS19*	A0A068VH99	3-ketoacyl-CoA synthase 19.
69	*CYTB5-E*	A0A068U9N1	Cytochrome b5 isoform E
70	*SUC4*	A0A068TXI0	Sucrose transport protein SUC4
71	*MCL19.11*	A0A068TVU7	Expressed protein.
72	*VIT1*	A0A068UD49	Vacuolar iron transporter 1
73	*F9K20.12*	A0A068UBR8	EP1-like glycoprotein 2.
74	*K18I23.5*	A0A068VKC8	AT5g05250/K18I23_5.
75	*MRO11.20*	A0A068UAL9	Copper transport protein family.
76	*F7H19.170*	A0A068UMR2	SPX domain-containing membrane protein At4g22990
77	*ZIF1*	A0A068UTC2	Protein ZINC INDUCED FACILITATOR 1
78	*RNS1*	A0A068TWK7	Ribonuclease 1
79	*CYP82C4*	A0A068UG96	Xanthotoxin 5-hydroxylase CYP82C4
80	*BHLH100*	A0A068ULW7	Transcription factor bHLH100
81	*COPT2*	A0A068UND2	Copper transporter 2
82	*ZIP8*	A0A068VA21	Probable zinc transporter 8
83	*PNC1*	A0A068V4C5	Peroxisomal adenine nucleotide carrier 1
84	*ZIP11*	A0A068V1J1	Zinc transporter 11
85	*PUB50*	A0A068TZT8	Putative U-box domain-containing protein 50
86	*AHA2*	A0A068U3T7	ATPase 2, plasma membrane-type
87	*F17A9.4*	A0A068TS39	Transmembrane protein.
88	*MP3*	A0A068VKG8	Probable steroid-binding protein 3.
89	*S8H*	A0A068V9Q2	Scopoletin 8-hydroxylase
90	*ECI1*	A0A068TXH7	Enoyl-CoA delta isomerase 1, peroxisomal
91	*PP2B15*	A0A068V9M8	F-box protein PP2-B15.

92	*ABCG37*	A0A068V3T5	ABC transporter G family member 37
93	*ECI2*	A0A068TSK9	Enoyl-CoA delta isomerase 2, peroxisomal
94	*CIPK11*	A0A068U8W3	CBL-interacting serine/threonine-protein kinase 11
95	*F12K2.6*	A0A068TLG3	GDSL esterase/lipase At2g27360.
96	*MQD19.19*	A0A068TS41	Aluminum induced protein with YGL and LRDR motifs.
97	*Q9LK01_ARATH*	A0A068U4D4	Alpha/beta-Hydrolases superfamily protein.
98	*F4IFT4_ARATH*	A0A068UEI3	Pollen Ole e 1 allergen and extensin family protein.
99	*F27G19.50*	A0A068UBF5	Aluminum induced protein with YGL and LRDR motifs.
100	*T17B22.4*	A0A068U090	Adenine nucleotide alpha hydrolases-like superfamily protein.
101	*PCO1*	A0A068U112	Plant cysteine oxidase 1
102	*ZIP3*	A0A068V8W7	Zinc transporter 3
103	*GLB3*	A0A068VD10	Two-on-two hemoglobin-3
104	*NQR-2*	A0A068VFF0	NADPH:quinone oxidoreductase
105	*AHB2*	A0A068TU61	Non-symbiotic hemoglobin 2
106	*NIA2*	A0A068VGB5	Nitrate reductase [NADH] 2
107	*F17F8.22*	A0A068VBI5	Molybdenum cofactor sulfurase family protein.
108	*POT2*	A0A068V074	Potassium transporter 2
109	*NPF6.3*	A0A068UNU3	Protein NRT1/ PTR FAMILY 6.3
110	*AKT2*	A0A068TNI0	Potassium channel AKT2/3
111	*ABCG25*	A0A068VAZ3	ABC transporter G family member 25.
112	*TCP20*	A0A068V859	Transcription factor TCP20
113	*NPF4.6*	A0A068TVL7	Protein NRT1/ PTR FAMILY 4.6

3. Supplementary Table S15. Phenylpropanoids biosynthesis

No	ID *A. thaliana*	ID *Coffea arabica*	ID *A. thaliana*
114	*SCPL17*	A0A068VMX7	Serine carboxypeptidase-like 17
115	*NUDT17*	A0A068V700	Nudix hydrolase 17, mitochondrial
116	*SCPL18*	A0A068VMX7	Serine carboxypeptidase-like 17
117	*PER43*	A0A068TN94	Peroxidase 43
118	*T6K12.21*	A0A068V8C1	Germin-like protein subfamily 1 member 3
119	*PER45*	A0A068U4B2	Peroxidase 45
120	*T29F13.9*	A0A068TU88	Pollen Ole e 1 allergen and extensin family protein.
121	*PER51*	A0A068U2Q0	Peroxidase 51
122	*MEE23*	A0A068UWE2	Berberine bridge enzyme-like 15
123	*PER52*	A0A068V211	Peroxidase 52
124	*PER11*	A0A068TW32	Peroxidase 11
125	*PER17*	A0A068TYW8	Peroxidase 17

126	*PER3*	A0A068VIB0	Peroxidase 3
127	*UGT72E1*	A0A068U3D1	UDP-glycosyltransferase 72E1
128	*CYP84A1*	A0A068U2U9	Cytochrome P450 84A1.
129	*ALDH2C4*	A0A068U6V3	Aldehyde dehydrogenase family 2 member C4
130	*SHT*	A0A068VKN8	Spermidine hydroxycinnamoyl transferase
131	*4CLL1*	A0A068U297	4-coumarate--CoA ligase-like 1
132	*4CLL7*	A0A068UET6	4-coumarate--CoA ligase-like 7
133	*CYP73A5*	A0A068VFV1	Trans-cinnamate 4-monooxygenase
134	*HST-2*	A0A068TQG5	Shikimate O-hydroxycinnamoyltransferase
135	*F12L6.8*	A0A068VH95	Alpha/beta-Hydrolases superfamily protein.
136	*F6'H2*	A0A068VH54	Feruloyl CoA ortho-hydroxylase 2
137	*MAP2B*	A0A068VEQ7	Methionine aminopeptidase 2B
138	*LAC16*	A0A068UDD6	Laccase-16
139	*AIG2LD*	A0A068UCN8	AIG2-like protein D
140	*LAC1*	A0A068TQR0	Laccase-1
141	*LAC3*	A0A068ULG7	Laccase-3
142	*CYP75B1*	A0A068VH53	Flavonoid 3'-monooxygenase
143	*A3G2XYLT*	A0A068UBT4	Anthocyanidin 3-O-glucoside 2'''-O-xylosyltransferase
144	*F24B9.7*	A0A068VEE8	Ribosomal protein L29 family protein.
145	*CHS*	A0A068VQ62	Chalcone synthase
146	*TT2*	A0A068TXJ6	Transcription factor TT2
147	*MYB21*	A0A068U0Q2	Transcription factor MYB21
148	*F11A3.17*	A0A068UDU4	Zinc finger CCCH domain-containing protein 21.
149	*GL3*	A0A068UUT4	Transcription factor GLABRA 3

4. Supplementary Table S16. Abscisic acid (ABA)

No	ID *A. thaliana*	ID *Coffea arabica*	ID *A. thaliana*
150	*UGT71C5*	A0A068UCG7	UDP-glycosyltransferase 71C5
151	*CYP707A1*	A0A068UHZ2	Abscisic acid 8'-hydroxylase 1
152	*SDR1*	A0A068US79	(+)-neomenthol dehydrogenase
153	*PYL11*	A0A068TLF4	Abscisic acid receptor PYL11
154	*PYL1*	A0A068UXN8	Abscisic acid receptor PYL1
155	*ABA2*	A0A068TSA0	Xanthoxin dehydrogenase
156	*F14D16.30*	A0A068U401	Ubiquinone biosynthesis protein
157	*LCY1*	A0A068UYZ4	Lycopene beta cyclase, chloroplastic
158	*VTE4*	A0A068VGE6	Tocopherol O-methyltransferase, chloroplastic
159	*FPS2*	A0A068UZP5	Farnesyl pyrophosphate synthase 2
160	*PSY1*	A0A068VNJ4	Phytoene synthase, chloroplastic
161	*SQE1*	A0A068TXL6	Squalene epoxidase 1

162	*CAS1*	A0A068UV98	Cycloartenol synthase
163	*3BE-TAHSD/D3*	A0A068U592	3beta-hydroxysteroid-dehydrogenase/decarboxylase isoform 3

5. Supplementary Table S17. AUX/IAA, STEM CELL and Cytokinin

No	ID *A. thaliana*	ID *Coffea arabica*	ID *A. thaliana*
164	*PPT1-2*	A0A068UV22	4-hydroxybenzoate polyprenyltransferase, mitochondrial
165	*CKX1*	A0A068V089	Cytokinin dehydrogenase 1
166	*LOG5*	A0A068V3Q5	Cytokinin riboside 5'-monophosphate phosphoribohydrolase
167	*CKX5*	A0A068UIL7	Cytokinin dehydrogenase 5
168	*AHK4*	A0A068UD10	Histidine kinase 4
169	*CKX3*	A0A068U3M8	Cytokinin dehydrogenase 3
170	*IPT3*	A0A068TSP3	Adenylate isopentenyltransferase 3, chloroplastic
171	*BRI1*	A0A068UX42	Protein BRASSINOSTEROID INSENSITIVE 1
172	*CAM7*	A0A068UMJ8	Calmodulin-7
173	*PCAP1*	A0A068U7F0	Plasma membrane-associated cation-binding protein 1
174	*BIM1*	A0A068TT57	Transcription factor BIM1
175	*CYP734A1*	A0A068U716	Cytochrome P450 734A1
176	*RKL1*	A0A068TSJ5	Probable inactive receptor kinase At1g48480
177	*SD17*	A0A068UB32	Receptor-like serine/threonine-protein kinase SD1-7
178	*ATHB-52*	A0A068TTU1	Homeobox-leucine zipper protein ATHB-52
179	*BRG3*	A0A068UYV3	Probable BOI-related E3 ubiquitin-protein ligase 3
180	*AHP1*	A0A068TSI8	Histidine-containing phosphotransfer protein 1
181	*WOX5*	A0A068V2D2	WUSCHEL-related homeobox 5
182	*SCR*	A0A068V5G6	Protein SCARECROW
183	*MGP*	A0A068V7J8	Zinc finger protein MAGPIE
184	*SCL3*	A0A068UJK6	Scarecrow-like protein 3
185	*SHR*	A0A068U370	Protein SHORT-ROOT
186	*CLV2*	A0A068U9G1	Receptor-like protein CLAVATA2
187	*PLT4*	A0A068V2A3	Probable polyol transporter 4
188	*IAA14*	A0A068TUY9	Auxin-responsive protein IAA14
189	*LBD16*	A0A068U6E5	LOB domain-containing protein 16
190	*WOX11*	A0A068VCF0	WUSCHEL-related homeobox 11
191	*WOX12*	A0A068VCF0	WUSCHEL-related homeobox 11
192	*ERF086*	A0A068TS67	Ethylene-responsive transcription factor ERF086
193	*NAC098*	A0A068UCE4	Protein CUP-SHAPED COTYLEDON 2
194	*JAG*	A0A068TX66	Zinc finger protein JAGGED
195	*WOX13*	A0A068TZT5	WUSCHEL-related homeobox 13

196	*YAB1*	A0A068VGH3	Axial regulator YABBY 1
197	*YAB5*	A0A068UPF7	Axial regulator YABBY 5
198	*HEC1*	A0A068VLN9	Transcription factor HEC1
199	*KAN1*	A0A068TZL4	Transcription repressor KAN1
200	*KAN4*	A0A068UG96	Xanthotoxin 5-hydroxylase CYP82C4
201	*KNAT6*	A0A068V806	Homeobox protein knotted-1-like 6
202	*BOB1*	A0A068U931	Protein BOBBER 1
203	*LSH4*	A0A068TV89	Protein LIGHT-DEPENDENT SHORT HYPOCOTYLS 4
204	*VCC*	A0A068UU57	GPI inositol-deacylase C, putative (DUF1218).
205	*MYB105*	A0A068TWL5	Transcription factor MYB105
206	*TAX1*	A0A068V5V7	Signaling peptide TAXIMIN 1

6. Supplementary Table S18. Response to auxin

No	ID *A. thaliana*	ID *Coffea arabica*	ID *A. thaliana*
207	*PTL*	A0A068V3X7	Trihelix transcription factor PTL
208	*AP3*	A0A068UG32	Floral homeotic protein APETALA 3
209	*FPF1*	A0A068TXC9	Flowering-promoting factor 1
210	*SOC1*	A0A068UKZ3	MADS-box protein SOC1
211	*PHYA*	A0A068VAD1	Phytochrome A
212	*AGL8*	A0A068VDS1	Agamous-like MADS-box protein AGL8
213	*SOT17*	A0A068U9D0	Cytosolic sulfotransferase 17
214	*SOT16*	A0A068TW30	Cytosolic sulfotransferase 16
215	*SOT18*	A0A068U9D0	Cytosolic sulfotransferase 17
216	*UGT74B1*	A0A068TY11	UDP-glycosyltransferase 74B1
217	*CYP83B1*	A0A068V616	Cytochrome P450 83B1
218	*ASB1*	A0A068UAA1	Anthranilate synthase beta subunit 1, chloroplastic
219	*CRYD*	A0A068VC81	Cryptochrome DASH, chloroplastic/mitochondrial
220	*HAT4*	A0A068UN80	Homeobox-leucine zipper protein HAT4
221	*IAA19*	A0A068TP25	Auxin-responsive protein IAA19
222	*IAA4*	A0A068TSZ6	Auxin-responsive protein IAA4
223	*EXPA11*	A0A068UFM0	Expansin-A11
224	*XTH15*	A0A068U5S0	Xyloglucan endotransglucosylase/hydrolase protein 15
225	*SAUR32*	A0A068TP82	Auxin-responsive protein SAUR32
226	*SAUR71*	A0A068UYT7	Auxin-responsive protein SAUR71
227	*MFB16.16*	A0A068UYJ4	SAUR-like auxin-responsive protein family.
228	*GH3.1*	A0A068U207	Probable indole-3-acetic acid-amido synthetase GH3.1
229	*T20N10_180*	A0A068VDD0	Haloacid dehalogenase (HAD) superfamily protein.
230	*NPC4*	A0A068VGT0	Non-specific phospholipase C4
231	*ACA4-2*	A0A068V1S3	Calcium-transporting ATPase 4, plasma membrane-type

232	*MXA21.22*	A0A068U573	#N/D
233	*GAD4*	A0A068UCQ2	Glutamate decarboxylase 1
234	*GAD1*	A0A068UCQ2	Glutamate decarboxylase 1
235	*ACO4*	A0A068U815	1-aminocyclopropane-1-carboxylate oxidase 4
236	*EBF2*	A0A068UQ79	EIN3-binding F-box protein 2
237	*ETR2*	A0A068TV52	Ethylene receptor 2
238	*ACS7*	A0A068U3B7	1-aminocyclopropane-1-carboxylate synthase 7
239	*F2P24.4*	A0A068UTN6	1-aminocyclopropane-1-carboxylate oxidase 5
240	*SAM2*	A0A068TS06	S-adenosylmethionine synthase 2
241	*SAMDC1*	A0A068U2H0	S-adenosylmethionine decarboxylase 1 alpha chain
242	*ACL5*	A0A068UYX5	Thermospermine synthase ACAULIS5
243	*PAO5*	A0A068U6C9	Probable polyamine oxidase 5

7. Supplementary Table S19. Supplementary table 13. Amino sugar and nucleotide sugar metabolism

No	ID *A. thaliana*	ID *Coffea arabica*	ID *A. thaliana*
244	*RGP1*	A0A068VH96	UDP-arabinopyranose mutase 1
245	*MUR4*	A0A068UVR3	UDP-arabinose 4-epimerase 1
246	*UXS2*	A0A068UEQ1	UDP-glucuronic acid decarboxylase 2
247	*NRS/ER*	A0A068UN06	Bifunctional dTDP-4-dehydrorhamnose 3,5-epimerase/dTDP-4-dehydrorhamnose reductase
248	*UGD3*	A0A068U2A1	UDP-glucose 6-dehydrogenase 3
249	*GAE1*	A0A068VL90	UDP-glucuronate 4-epimerase 1
250	*GAE6*	A0A068VMP1	UDP-glucuronate 4-epimerase 6
251	*TRE1*	A0A068UUM1	Trehalase
252	*HPR*	A0A068V1N1	Glycerate dehydrogenase HPR, peroxisomal
253	*GLO4*	A0A068TNM6	Peroxisomal (S)-2-hydroxy-acid oxidase GLO4.
254	*STP7*	A0A068UQ08	Sugar transport protein 7
255	*CICDH*	A0A068U289	Cytosolic isocitrate dehydrogenase [NADP]
256	*ICL*	A0A068UTQ7	Isocitrate lyase
257	*PCBER1*	A0A068TM70	Phenylcoumaran benzylic ether reductase 1
258	*XTH8*	A0A068TU18	Probable xyloglucan endotransglucosylase/hydrolase protein 8
259	*F19C24.7*	A0A068TNM5	Amino acid dehydrogenase family protein

8. Supplementary Table S20. Beta-glucosidase activity

No	ID *A. thaliana*	ID *Coffea arabica*	ID *A. thaliana*
260	*T22P22.110*	A0A068UW10	Glycosyl hydrolases family 31 protein
261	*BGLU41*	A0A068UCA6	Putative beta-glucosidase 41.
262	*BGLU44*	A0A068TSQ1	Beta-glucosidase 44
263	*BGLU46*	A0A068TTX5	Beta-glucosidase 46
264	*BGLU17*	A0A068UH00	Beta-glucosidase 17
265	*BGLU40*	A0A068TWI4	Beta-glucosidase 40.
266	*BGLU11*	A0A068VIH4	Beta-glucosidase 11
267	*BGLU12*	A0A068UHP9	Beta-glucosidase 12.
268	*BGLU13*	A0A068UHP9	Beta-glucosidase 12.
269	*BGLU47*	A0A068TTX5	Beta-glucosidase 46
270	*BAM1-2*	A0A068VIA8	Beta-amylase 1, chloroplastic
271	*BFRUCT3*	A0A068UN82	Acid beta-fructofuranosidase 3, vacuolar
272	*T22P22_160*	A0A068UI96	NADH dehydrogenase [ubiquinone] iron-sulfur protein 7, mitochondrial
273	*Non homologue*	YMF19	
274	*Non homologue*	COX3	
275	*Non homologue*	COB	
276	*ATP9*	A0A068UT96	ATP synthase subunit 9, mitochondrial
277	C/VIF1	A0A068TR99	Cell wall / vacuolar inhibitor of fructosidase 1
278	*Q9LW11_ARATH*	A0A068U8I2	Zinc finger protein-like Ser/Thr protein kinase-like protein.
279	*MLP28*	A0A068VLE8	MLP-like protein 28
280	*SKS6*	A0A068UFT0	SKU5-similar 6.
281	*ENODL17*	A0A068U167	Lamin-like protein.
282	*ENODL8*	A0A068UUN2	Early nodulin-like protein 8.
283	*MMI9.18*	A0A068UA16	Ripening-related protein-like
284	*F10B6.30*	A0A068TXN8	Plant invertase/pectin methylesterase inhibitor superfa-mily protein.
285	*PMEI9*	A0A068UQL1	Pectinesterase inhibitor 9
286	*XYL1*	A0A068TWI1	Alpha-xylosidase 1
287	*F21O3.3*	A0A068U5Z2	Putative glucan endo-1-3-beta-glucosidase
288	*K9L2.20*	A0A068UWE2	Berberine bridge enzyme-like 26.
289	*F12K11.24*	A0A068TTF6	1-aminocyclopropane-1-carboxylate oxidase homolog 1.
290	*petC*	A0A068UUF3	Cytochrome b6-f complex iron-sulfur subunit, chloro-plastic
291	*CWINV1*	A0A068TPN4	Beta-fructofuranosidase, insoluble isoenzyme CWINV1
292	*WRKY27*	A0A068U994	Probable WRKY transcription factor 27
293	*AMY1*	A0A068TZJ3	Alpha-amylase 1

294	*AGAL2*	A0A068VBL5	Alpha-galactosidase 2
295	*HCF173*	A0A068VG30	Protein HIGH CHLOROPHYLL FLUORESCENCE PHENOTYPE 173, chloroplastic
296	*RFS6*	A0A068UQA6	Probable galactinol--sucrose galactosyltransferase 6
297	*F8M21.30*	A0A068U1R0	Galactose mutarotase-like superfamily protein.
298	*F14G24.14*	A0A068UBL6	Peroxisomal membrane 22 kDa (Mpv17/PMP22) family protein.
299	*F4F7.37*	A0A068UB90	Putative clathrin assembly protein At1g25240.
300	*T23J7.130*	A0A068UVN7	Aldose 1-epimerase
301	*AGAL1*	A0A068V7X5	Alpha-galactosidase 1
302	*F15N18.10*	A0A068UAY5	Uncharacterized protein At5g11420.

9. Supplementary Table S21. Glutathione metabolism

No	ID *A. thaliana*	ID *Coffea arabica*	ID *A. thaliana*
303	*GSTL3*	A0A068U7Q6	Glutathione S-transferase L3
304	*GSTT1*	A0A068UMV0	Glutathione S-transferase T1
305	*GSTT2*	A0A068UMV0	Glutathione S-transferase T1
306	*GSTU10*	A0A068VIP8	Glutathione S-transferase U10
307	*GSTU17*	A0A068TY44	Glutathione S-transferase U17
308	*GSTU19*	A0A068VJ79	Glutathione S-transferase U19
309	*GSTU25*	A0A068VJ79	Glutathione S-transferase U19
310	*GSTU8*	A0A068UFZ4	Glutathione S-transferase U8
311	*GSTU9*	A0A068VIP8	Glutathione S-transferase U10
312	*GPX6*	A0A068UNF2	Probable phospholipid hydroperoxide glutathione peroxidase 6, mitochondrial
313	*F1E22.17*	A0A068U9T6	Microsomal glutathione s-transferase.
314	*CYP76C2*	A0A068VB47	Cytochrome P450 76C2
315	*CYP72A14*	A0A068V5N2	Cytochrome P450 72A14
316	*F1N19.7*	A0A068UUK6	Putative peptide transporter protein.
317	*CYP71B10*	A0A068V616	Cytochrome P450 71B10

10. Supplementary Table S22. Cell wall biogenesis

No	ID *A. thaliana*	ID *Coffea arabica*	ID *A. thaliana*
318	*T5I8.15*	A0A068UWE8	Berberine bridge enzyme-like 8.
319	*SAP12*	A0A068U0X2	Zinc finger AN1 domain-containing stress-associated protein 12
320	*PME17*	A0A068VJU0	Probable pectinesterase/pectinesterase inhibitor 17

321	*F13H10.7*	A0A068ULS9	S-adenosyl-L-methionine-dependent methyltransferases superfamily protein.
322	*PGIP1*	A0A068UNJ9	Polygalacturonase inhibitor 1
323	*F17A9.16*	A0A068U5L4	Probable pectate lyase 8.
324	*F15K9.16*	A0A068UTA4	Eukaryotic aspartyl protease family protein
325	*MDC12_15*	A0A068V348	Probable pectate lyase 22.
326	*F12A21.12*	A0A068UYG3	Probable pectate lyase 5.
327	*PME26*	A0A068TR01	Putative pectinesterase/pectinesterase inhibitor 26
328	*PME4*	A0A068V8Q6	Pectinesterase 4
329	*PME33*	A0A068VJK2	Probable pectinesterase/pectinesterase inhibitor 33
330	*PME2*	A0A068V3C2	Pectinesterase 2
331	*PME20*	A0A068VBG7	Probable pectinesterase/pectinesterase inhibitor 20
332	*A0A1I9LP65*	A0A068UIN7	Pectin lyase-like superfamily protein.
333	*DTX14*	A0A068TWR4	Protein DETOXIFICATION 14.
334	*BXL4*	A0A068TNE2	Beta-D-xylosidase 4
335	*BXL7*	A0A068TY76	Probable beta-D-xylosidase 7.
336	*F17A17.37*	A0A068UJL5	F17A17.37 protein.
337	*PUB21*	A0A068TZE1	U-box domain-containing protein 21
338	*T11A7.9*	A0A068VLY9	Uncharacterized protein At2g41810.

10. Supplementary Table S23. Jasmonic acid signaling

No	ID *A. thaliana*	ID *Coffea arabica*	ID *A. thaliana*
339	*ERF1B*	A0A068TT86	Ethylene-responsive transcription factor 1B
340	*EP3*	A0A068UT78	Endochitinase EP3
341	*CYP74A*	A0A068UIY6	Allene oxide synthase, chloroplastic.
342	*CHIB1*	A0A068ULJ1	Acidic endochitinase
343	*ChiC*	A0A068UNX2	Class V chitinase
344	*CYP71B23*	A0A068UWD9	Cytochrome P450 71B23
345	*KTI3*	A0A068VF63	Kunitz trypsin inhibitor 3
346	*NAC056*	A0A068UE09	NAC transcription factor 56
347	*SD25*	A0A068UYL7	G-type lectin S-receptor-like serine/threonine-protein kinase SD2-5
348	*PP2-A10*	A0A068US48	Phloem protein 2-A10.
349	*AVT1A*	A0A068VM61	Amino acid transporter AVT1A.
350	*DMR6*	A0A068UBG5	Protein DOWNY MILDEW RESISTANCE 6
351	*F2J10.6*	A0A068TPM9	F2J10.6 protein
352	*HEL*	A0A068VJ77	Hevein-like preproprotein
353	*OPR2*	A0A068UR82	12-oxophytodienoate reductase 2
354	*VSP1*	A0A068U9Z4	Vegetative storage protein 1

355	*LOX1*	A0A068V4P9	Linoleate 9S-lipoxygenase 1
356	*PRB1*	A0A068TPM9	F2J10.6 protein
357	*OSM34*	A0A068UMX5	Osmotin-like protein OSM34
358	*RPM1*	A0A068VEE2	Disease resistance protein RPM1
359	*SCL14*	A0A068TVQ0	Scarecrow-like protein 14
360	*RPP13L4*	A0A068U0D6	Disease resistance RPP13-like protein 4
361	*ZED1*	A0A068UU04	Non-functional pseudokinase ZED1
362	*AT5G12460.1*	A0A068UKW8	Fringe-like protein (DUF604).
363	*BZIP34*	A0A068V5U8	Basic leucine zipper 34
364	*BZIP9*	A0A068VGH1	Basic leucine zipper 9
365	*TGA10*	A0A068UPD7	Transcription factor TGA10
366	*GRXC9*	A0A068V524	Glutaredoxin-C9
367	*HEXO2*	A0A068UJ57	Beta-hexosaminidase 2
368	NPR4	A0A068VA17	Regulatory protein NPR4
369	GSTF8	A0A068U7C7	Glutathione S-transferase F8
370	GGR	A0A068VMV1	Heterodimeric geranylgeranyl pyrophosphate synthase small subunit
371	CER1	A0A068UM94	Very-long-chain aldehyde decarbonylase CER1
372	CHI-B	A0A068U0X4	Basic endochitinase B
373	F22D1.120	A0A068V529	Glycosyl hydrolase family protein.
