# Supplementary material for: Transcriptomic Analysis of Osmotic Stress-Tolerant Somatic Embryos of Coffea arabica L. Mediated by the Coffee Antisense Trehalase Gene: A Marker-Free Approach
Source: Int J Mol Sci. 2025 Sep 21;26(18):9224. doi: 10.3390/ijms26189224 (PMC12471068; doi:10.3390/ijms26189224)
Supplement: Supplementary file 1 [file ijms-26-09224-s001.zip › Supplementary Table S24 PRIMERs -plants.pdf]

Article

Supplementary Table S24 Materials and Methods

Transcriptomic analysis of osmotic stress tolerant somatic embryos of *Coffea arabica* L. mediated by the coffee antisense *Trehalase* gene: A Marker free approach.

Eliana Valencia-Lozano[1*], Aarón Barraza[2], Jorge Ibarra[3], John P. Délano-Frier[3], Norma Martínez-Gallardo[3], Anali Gamez-Escobedo[4] and José Luis Cabrera-Ponce[5*]

1. Primer Validation transcriptome

No	*A. thaliana*	String	ID C. arabica	*ID C. arabica*	Forward	Reverse
1	*TPPB*	A0A068TXJ5	XP_027091185	*XM_027235384.1*	CCGCAATAGTTACCGGAAGATG	GTTGGTCCCTTGATGTCCATAC
2	*P5CSB*	A0A068TXS1	XP_027110917.1	*XM_027255116.1*	GGGAGCTATTACCTTCGGAAAC	AACAGGGATGCCAGCATAAG
3	*SERK1*	A0A068TXX7	XP_027094258	*XM_027238457.1*	GGAGCTGGTGGGTTAAGAAA	CCAGAGCACCACTGAAACTAA
4	*TPPD*	A0A068TYT9	XP_027074014	*XM_027218213.1*	CTTCTGACGACCAATCCTCTTC	CGTCGTAGTCGAGAAACATCAC
5	*PHS1-3*	A0A068U3V8	XP_027074767.1	*XM_027218966.1*	ATACCTCTCCCAGCTCAAATTC	GGGAGGATGCAATACACAGTTA
6	*BBM*	A0A068U6P3	XP_027062561.1	*XM_027206760.1*	GCTGCATGGGTTGATGAATATG	TCCCAAGTACCCTCCACTATAA
7	*NFYB6*	A0A068U7K3	XP_027085797	*XM_027229996.1*	CTGGGAGATCTGATGGAACTTG	GCTTATCTCTGCTGCTCTCATC
8	*ABI3*	A0A068U8A0	XP_027071287.1	*XM_027215486.1*	GATGGCGGGCTATACTTTCTAC	AGAGCGCTTGAGCTTGATAC
9	*VAL2*	A0A068UCW0	XP_027069391.1	*XM_027213590.1*	CCAAAGAAGCCCAGGAAGATAC	CCATTTCATTCCTTCCCGAGAG
10	*CDC48A*	A0A068UGL5	XP_027085377.1	*XM_027229576.1*	GTCCACTTCTCGCTTGTTATCT	CGTCTTCGGATTTCCCTCTTT
11	*WOX2*	A0A068UL49	XP_027126371.1	*XM_027270570.1*	CCCGACAAAGGAGCAAATAGA	CCATAAGCCCTTAGCCTGTTAG
12	*PCNA2*	A0A068UNF1	XP_027089310.1	*XM_027233509.1*	CTACCGCTGTGACCGTAATTT	TGCCATCGTCGCCTTTAAT
13	*TRE1*	A0A068UUM1	XP_027085642	*XM_027229841.1*	CTGGCTTGCTGAGACCAATA	TGACCCACAAGGGAATGAAG
14	*AGL15*	A0A068V010	XP_027069013.1	*XM_027213212.1*	CAGGCAAGTCACCTTCTCAA	ACCTCTGCATCGCACAAA
15	*ENO2*	A0A068V643	XP_027072612.1	*XM_027216811.1*	GTTTGGCAACGGGTCAAATC	ATCTGCACCAAGTTCCTCTTC

16	*FUS3*	A0A068V7Y1	XP_027102113.1	*XM_027246312.1*	GGAAGTAGCAGTGGTGGTAATG	ACCGCTTCACTCTGGAAATC
17	*MCM7*	A0A068V8F7	XP_027118445.1	*XM_027262644.1*	CCTGATCCTTCTTCGGGAATTT	TCACGACAGCACACATACTAAC

Endogenous genes:

C. arabica	NCBI	Forward	Reverse
24S	GT730897.1	GACCAATCGTCTTCTTTCCAGAAA	TCAACTCAGCCTTGGAAACATTAG
RPL39	GT720707.1	GCGAAGAAGCAGAGGCAGAA	TTGGCATTGTAGCGGATGGT

- Endogenous by [90] Freitas, N.C.; Barreto, H.G.; Fernandes-Brum, C.N., Moreira, R.O.; Chalfun-Junior, A. & Paiva, L.V. Validation of reference genes for qPCR analysis of *Coffea arabica* L. somatic embryogenesis-related tissues. *Plant Cell, Tissue and Organ Culture (PCTOC)* **2017**, 128, 663-678.
