# Supplementary material for: Transcriptomic Analysis of Osmotic Stress-Tolerant Somatic Embryos of Coffea arabica L. Mediated by the Coffee Antisense Trehalase Gene: A Marker-Free Approach
Source: Int J Mol Sci. 2025 Sep 21;26(18):9224. doi: 10.3390/ijms26189224 (PMC12471068; doi:10.3390/ijms26189224)
Supplement: Supplementary file 1 [file ijms-26-09224-s001.zip › Supplementary Tables S2 to S11 UPREGULATED.pdf]

Article

Supplementary Tables S2 to S11

Transcriptomic analysis of osmotic stress tolerant somatic embryos of *Coffea arabica* L. mediated by the coffee antisense *Trehalase* gene: A Marker free approach.

Eliana Valencia-Lozano[1]*, Aarón Barraza[2], Jorge Ibarra[3], John P. Délano-Frier[3], Norma Martínez-Gallardo[3], Anali Gamez-Escobedo[4] and José Luis Cabrera-Ponce[5]*

1. Supplementary Table S2. Somatic Embryogenesis

No	ID *A. thaliana*	ID *Coffea arabica*	Associated function
1	*ABI3*	*A0A068U8A0*	B3 domain-containing transcription factor ABI3
2	*AGL15*	*A0A068V010*	Agamous-like MADS-box protein AGL15
3	*AHK5*	*A0A068V1M6*	Histidine kinase 5
4	*ARR4*	*A0A068TWC5*	Two-component response regulator ARR4
5	*AUX1*	*A0A068UD59*	Auxin transporter protein 1
6	*BBM*	*A0A068U6P3*	AP2-like ethylene-responsive transcription factor BBM
7	*CDC48A*	*A0A068UGL5*	Cell division control protein 48 homolog A
8	*EM1*	*A0A068UD22*	Em-like protein GEA1
9	*EM6*	*A0A068V633*	Em-like protein GEA6
10	*F22M8.6*	*A0A068VAP1*	Zinc finger protein-like protein.
11	*FUS3*	*A0A068V7Y1*	B3 domain-containing transcription factor FUS3
12	*GA3OX2*	*A0A068VEF1*	Gibberellin 3-beta-dioxygenase 2
13	*LEA46*	*A0A068UPG4*	Late embryogenesis abundant protein 46
14	*NF-YC13*	*A0A068V0C8*	Nuclear factor Y, subunit C13.
15	*NFYA2*	*A0A068UPW6*	Nuclear transcription factor Y subunit A-2
16	*NFYA5*	*A0A068TPA3*	Nuclear transcription factor Y subunit A-5
17	*NFYA6*	*A0A068UH68*	Nuclear transcription factor Y subunit A-6
18	*NFYA7*	*A0A068V9V1*	Nuclear transcription factor Y subunit A-7
19	*NFYA8*	*A0A068VIL0*	Nuclear transcription factor Y subunit A-8
20	*NFYA9*	*A0A068VE79*	Nuclear transcription factor Y subunit A-9
21	*NFYB6*	*A0A068U7K3*	Nuclear transcription factor Y subunit B-6
22	*NFYB9*	*A0A068UXD0*	Nuclear transcription factor Y subunit B-9
23	*PI4KG4*	*A0A068UM62*	Phosphatidylinositol 4-kinase gamma 4
24	*SERK1*	*A0A068TXX7*	Somatic embryogenesis receptor kinase 1
25	*UFD1*	*A0A068TNP5*	Ubiquitin fusion degradation 1.

26	*VAL2*	*A0A068UCW0*	B3 domain-containing transcription repressor VAL2
27	*WOX2*	*A0A068UL49*	WUSCHEL-related homeobox 2
28	*UBP14*	*A0A068UBB6*	Ubiquitin carboxyl-terminal hydrolase 14
29	*RLK5*	*A0A068TVW5*	Receptor-like protein kinase 5
30	*RUB1*	*A0A068V111*	Ubiquitin-NEDD8-like protein RUB1

2. Supplementary Table S3. Cell Cycle

No	**ID *A. thaliana***	**ID *Coffea arabica***	**Associated function**
31	*ATIM*	*A0A068UBU6*	Timeless family protein.
32	*B3LFC1_ARATH*	*A0A068U1E4*	Exonuclease family protein.
33	*BARD1*	*A0A068U850*	BRCA1-associated RING domain protein 1
34	*BRCA2B*	*A0A068UCZ6*	Protein BREAST CANCER SUSCEPTIBILITY 2 homolog B
35	*BUBR1*	*A0A068UFL7*	Mitotic spindle checkpoint protein BUBR1
36	*CAPH2*	*A0A068TRA1*	Condensin-2 complex subunit H2
37	*CDC6*	*A0A068UNK9*	Cell division control protein 6 homolog
38	*CDKB1-1*	*A0A068VN88*	Cyclin-dependent kinase B1-1
39	*CDKB1-2*	*A0A068UDI8*	Cyclin-dependent kinase B1-2
40	*CDKB2-1*	*A0A068UR97*	Cyclin-dependent kinase B2-1
41	*CDKB2-2*	*A0A068UDI8*	Cyclin-dependent kinase B1-2
42	*CDT1A*	*A0A068U5J0*	CDT1-like protein a, chloroplastic
43	*CTC1*	*A0A068VHN1*	CST complex subunit CTC1
44	*CTF18*	*A0A068TT40*	P-loop containing nucleoside triphosphate hydrolases superfamily protein.
45	*CYCA1-1*	*A0A068VFE6*	Cyclin-A1-1
46	*CYCA1-2*	*A0A068URS1*	Cyclin-A1-2
47	*CYCA2-3*	*A0A068TQ86*	Cyclin-A2-3
48	*CYCB1-4*	*A0A068UN73*	Cyclin-B1-4
49	*CYCB2-3*	*A0A068TZY4*	Cyclin-B2-3.
50	*CYCB2-4*	*A0A068UG94*	Cyclin-B2-4.
51	*CYCB3-1*	*A0A068UZ80*	Putative cyclin-B3-1
52	*CYCD5-1*	*A0A068U246*	Cyclin-D5-1.
53	*CYCD7-1*	*A0A068UE90*	Putative cyclin-D7-1
54	*dl3985w*	*A0A068TSB2*	Condensin-2 complex subunit.
55	*DPB2*	*A0A068TTG1*	DNA polymerase epsilon subunit B
56	*EMB2656*	*A0A068U0F3*	ARM repeat superfamily protein.
57	*ESP1*	*A0A068TNC3*	Separase
58	*ETG1*	*A0A068UZR6*	Mini-chromosome maintenance complex-binding protein
59	*EXO1*	*A0A068V235*	Exonuclease 1
60	*F2D10.21*	*A0A068VKU9*	RAD3-like DNA-binding helicase protein.

61	*F5A8.9*	*A0A068U440*	Zinc finger (C3HC4-type RING finger) family protein / BRCT domain-containing protein.
62	*FZR3*	*A0A068TVY7*	Protein FIZZY-RELATED 3
63	*K20J1.8*	*A0A068TRI5*	Fanconi anemia group I-like protein.
64	*KIN10C*	*A0A068UAJ9*	Kinesin-like protein KIN-10C
65	*KIN14G*	*A0A068V961*	Kinesin-like protein KIN-14G
66	*KIN14R*	*A0A068TZW2*	Kinesin-like protein KIN-14R.
67	*KIN4C*	*A0A068UTB9*	Kinesin-like protein KIN-4C
68	*KIN5C*	*A0A068V4J1*	Kinesin-like protein KIN-5C
69	*MAP65-3*	*A0A068U9S3*	65-kDa microtubule-associated protein 3
70	*MCM2*	*A0A068VDS9*	DNA replication licensing factor MCM2
71	*MCM3*	*A0A068VIW0*	DNA replication licensing factor MCM3
72	*MCM4*	*A0A068TNS0*	DNA replication licensing factor MCM4
73	*MCM5*	*A0A068V150*	DNA replication licensing factor MCM5
74	*MCM6*	*A0A068U916*	DNA replication licensing factor MCM6
75	*MCM7*	*MCM7-2*	DNA replication licensing factor MCM7
76	*MCM8*	*A0A068UGH6*	Probable DNA helicase MCM8
77	*MCM9*	*A0A068TN78*	Probable DNA helicase MCM9
78	*MER3*	*A0A068U1N4*	DExH-box ATP-dependent RNA helicase DExH17
79	*MRH10.12*	*A0A068U5N4*	Fanconi anemia group F protein (FANCF).
80	*MS5*	*A0A068UVJ7*	Protein POLLENLESS 3
81	*MSH2*	*A0A068TQC7*	DNA mismatch repair protein MSH2
82	*MUS81*	*A0A068V703*	Crossover junction endonuclease MUS81
83	*ORC5*	*A0A068UMG4*	Origin of replication complex subunit 5
84	*PCH2*	*A0A068UW34*	Pachytene checkpoint protein 2 homolog
85	*PCNA2*	*A0A068UNF1*	Proliferating cell nuclear antigen 2
86	*POLA*	*A0A068U1V9*	DNA polymerase alpha catalytic subunit
87	*POLA2*	*A0A068UXQ0*	DNA polymerase alpha subunit B
88	*POLA3*	*A0A068UV29*	DNA primase
89	*PYM*	*A0A068UNI9*	Protein POLYCHOME
90	*RECQL1*	*A0A068VBY6*	ATP-dependent DNA helicase Q-like 1
91	*RECQL3*	*A0A068U3I1*	ATP-dependent DNA helicase Q-like 3
92	*RECQL4A*	*A0A068TQC9*	ATP-dependent DNA helicase Q-like 4A
93	*RECQL4B*	*A0A068VDG6*	ATP-dependent DNA helicase Q-like 4B
94	*RECQL5*	*A0A068UXQ4*	ATP-dependent DNA helicase Q-like 5
95	*RMI1*	*A0A068USI5*	RecQ-mediated genome instability protein 1
96	*RPA1B*	*A0A068VHF0*	Replication protein A 70 kDa DNA-binding subunit B
97	*RPA1D*	*A0A068V2B3*	Replication protein A 70 kDa DNA-binding subunit D
98	*RPA3B*	*A0A068V4C9*	Replication protein A 14 kDa subunit B
99	*SMC2-1*	*A0A068V0V7*	Structural maintenance of chromosomes protein 2-1
100	*SYN3*	*A0A068UJE9*	Sister chromatid cohesion 1 protein 3

101	*T10F20.10*	*A0A068TR00*	5'-3' exonuclease family protein.
102	*T22A6.170*	*A0A068TU74*	Phosphorylase superfamily protein.
103	*T32B20.70*	*A0A068V4K7*	ATP-dependent DNA helicase
104	*TK1A*	*A0A068VCH1*	Thymidine kinase a

3. Supplementary Table S4. Chromatin Remodeling

No	**ID *A. thaliana***	**ID *Coffea arabica***	**Associated function**
105	*AGO1*	*A0A068TRH0*	Protein argonaute 1
106	*AGO4*	*A0A068VGY8*	Protein argonaute 4
107	*AGO9*	*A0A068UN09*	Protein argonaute 9
108	*CYP40*	*A0A068TLQ8*	Peptidyl-prolyl cis-trans isomerase CYP40
109	*DCL2*	*A0A068U6K0*	Endoribonuclease Dicer homolog 2
110	*DRD1*	*A0A068TLA1*	Protein CHROMATIN REMODELING 35
111	*FDM1*	*A0A068TQN5*	Factor of DNA methylation 1
112	*H2AXA*	*A0A068U7I1*	Probable histone H2AXa
113	*H2B*	*A0A068UKE3*	Histone H2B.6
114	*HDA15*	*A0A068TQC4*	Histone deacetylase 15
115	*HDA5*	*A0A068VES0*	Histone deacetylase 5
116	*HTA12*	*A0A068UEG5*	Probable histone H2A.4
117	*HTA6*	*A0A068UL21*	Probable histone H2A.7
118	*HTB4*	*A0A068UKK4*	Histone H2B.11
119	*HTR2*	*A0A068USU6*	Histone H3.2
120	*JMJ25*	*A0A068VEN2*	Lysine-specific demethylase JMJ25
121	*RDR1*	*A0A068UAS7*	RNA-dependent RNA polymerase 1
122	*RDR2*	*A0A068VES3*	RNA-dependent RNA polymerase 2
123	*RDR5*	*A0A068U4Q5*	Probable RNA-dependent RNA polymerase 5
124	*SDE3*	*A0A068TS04*	Probable RNA helicase SDE3
125	*SUVH4*	*A0A068V7Y8*	Histone-lysine N-methyltransferase, H3 lysine-9 specific SUVH4
126	*T11P11.3*	*A0A068UG92*	Histone H2B.3
127	*T23G18.3*	*A0A068TU56*	Histone H2B.2
128	*TSK*	*A0A068UWU1*	Protein TONSOKU
129	*DMS3*	*A0A068U7L9*	Protein DEFECTIVE IN MERISTEM SILENCING 3
130	*ORTH2*	*A0A068ULA9*	E3 ubiquitin-protein ligase ORTHRUS 2
131	*CMT3*	*A0A068UZW0*	DNA (cytosine-5)-methyltransferase CMT3
132	*DDB2*	*A0A068ULV6*	Protein DAMAGED DNA-BINDING 2
133	*DDM1*	*A0A068U338*	ATP-dependent DNA helicase DDM1
134	*DRS1*	*A0A068V1A7*	DROUGHT SENSITIVE 1.
135	*EMB2271*	*A0A068UBQ4*	U3 snoRNP-associated protein-like EMB2271

136	*FAS2*	*A0A068U875*	Chromatin assembly factor 1 subunit FAS2
137	*PPAN*	*A0A068UVI5*	Peter Pan-like protein

4. Supplementary Table S5. Trehalose biosynthesis

No	**ID *A. thaliana***	**ID *Coffea arabica***	**Associated function**
138	*CFBP1*	*A0A068UD94*	Fructose-1,6-bisphosphatase 1, chloroplastic
139	*DXS*	*A0A068TRJ4*	1-deoxy-D-xylulose-5-phosphate synthase, chloroplastic
140	*SUS6*	*A0A068TZQ1*	Sucrose synthase 6
141	*T16L24.30*	*A0A068U357*	Probable fructokinase-4
142	*T22P22.110*	*A0A068UW10*	Glycosyl hydrolases family 31 protein
143	*TPPA*	*A0A068U9V1*	Trehalose-phosphate phosphatase A
144	*TPPB*	*A0A068TXJ5*	Trehalose-phosphate phosphatase B
145	*TPPD*	*A0A068TYT9*	Probable trehalose-phosphate phosphatase D
146	*TPPG*	*A0A068U944*	Probable trehalose-phosphate phosphatase G

5. Supplementary Table S6. Carbon Metabolism

No	**ID *A. thaliana***	**ID *Coffea arabica***	**Associated function**
147	*DUT*	*A0A068UG29*	Deoxyuridine 5'-triphosphate nucleotidohydrolase
148	*ENO2*	*A0A068V643*	Bifunctional enolase 2/transcriptional activator
149	*ENO3*	*A0A068ULH0*	Cytosolic enolase 3.
150	*F16L1.10*	*A0A068TYM2*	Phosphoglycerate mutase family protein.
151	*GDH1*	*A0A068UZB5*	Glycine cleavage system H protein 1, mitochondrial
152	*PGDH1*	*A0A068TM02*	D-3-phosphoglycerate dehydrogenase 1, chloroplastic
153	*PHS1-3*	*A0A068U3V8*	Alpha-glucan phosphorylase 1
154	*PKP1*	*A0A068TS59*	Plastidial pyruvate kinase 1, chloroplastic
155	*THY-1*	*A0A068UIN3*	Bifunctional dihydrofolate reductase-thymidylate synthase 1

6. Supplementary Table S7. Oxidative Stress

No	**ID *A. thaliana***	**ID *Coffea arabica***	**Associated function**
156	*APX1*	*A0A068UMU7*	L-ascorbate peroxidase 1, cytosolic
157	*APX2*	*A0A068UGH3*	L-ascorbate peroxidase 2, cytosolic
158	*ATJ8*	*A0A068TPL8*	Chaperone protein dnaJ 8, chloroplastic
159	*CAT2*	*A0A068U2G9*	Catalase-2

160	*GOLS1*	*A0A068V3M2*	Galactinol synthase 1
161	*GOLS3*	*A0A068V5V2*	Galactinol synthase 3
162	*GOLS4*	*A0A068UY65*	Galactinol synthase 4
163	*HSP17.6A*	*A0A068UKZ1*	17.6 kDa class I heat shock protein 1
164	*HSP70-2*	*A0A068UKG2*	Heat shock 70 kDa protein 2
165	*HSP70-4*	*A0A068UKG5*	Heat shock 70 kDa protein 4
166	*RFS5*	*A0A068U1M9*	Probable galactinol--sucrose galactosyltransferase 5
167	*TDX*	*A0A068TQ11*	TPR repeat-containing thioredoxin TDX

7. Supplementary Table S8. Phenilpropanoid biosynthesis

No	**ID *A. thaliana***	**ID *Coffea arabica***	**Associated function**
168	*4CL1*	*A0A068TPN7*	4-coumarate--CoA ligase 1
169	*4CLL6*	*A0A068UTX2*	4-coumarate--CoA ligase-like 6
170	*4CLL7*	*A0A068UET6*	4-coumarate--CoA ligase-like 7
171	*ABCG37*	*A0A068V3T5*	ABC transporter G family member 37
172	*CAD7*	*A0A068TZ70*	Cinnamyl alcohol dehydrogenase 7
173	*CCOAOMT1*	*CCoAOMT1*	Caffeoyl-CoA O-methyltransferase 1
174	*CCR1-2*	*A0A068TSC2*	Cinnamoyl-CoA reductase 1
175	*CSE*	*A0A068TPC0*	Caffeoylshikimate esterase
176	*CYP73A5*	*A0A068VFV1*	Trans-cinnamate 4-monooxygenase
177	*CYP75B1*	*A0A068VH53*	Flavonoid 3'-monooxygenase
178	*CYP82C4*	*A0A068UG96*	Xanthotoxin 5-hydroxylase CYP82C4
179	*CYP98A3*	*A0A068VEU9*	Cytochrome P450 98A3
180	*F4IZK0_ARATH*	*A0A068VBS4*	Alpha/beta-Hydrolases superfamily protein.
181	*F6'H1*	*A0A068VH54*	Feruloyl CoA ortho-hydroxylase 1
182	*HST-2*	*A0A068TQG5*	Shikimate O-hydroxycinnamoyltransferase
183	*LAC17*	*A0A068TZK5*	Laccase-17
184	*PAL1*	*A0A068VM15*	Phenylalanine ammonia-lyase 1
185	*PER71*	*A0A068TQ20*	Peroxidase 71
186	*S8H*	*A0A068V9Q2*	Scopoletin 8-hydroxylase
187	*UGT72E1*	*A0A068U3D1*	UDP-glycosyltransferase 72E1
188	*F14A1.10*	*A0A068UM02*	Putative acyl-activating enzyme 19

8. Supplementary Table S9. Glucosinolates, Strictosidine biosynthesis

No	**ID *A. thaliana***	**ID *Coffea arabica***	**Associated function**
189	*LOX3*	*A0A068VLV0*	Lipoxygenase 3, chloroplastic
190	*P5CSB*	*A0A068TXS1*	Delta-1-pyrroline-5-carboxylate synthase B

191	*APK1*	*A0A068V0Y1*	Adenylyl-sulfate kinase 1, chloroplastic
192	*APK2*	*A0A068U2S6*	Adenylyl-sulfate kinase 2, chloroplastic
193	*SOT17*	*A0A068U9D0*	Cytosolic sulfotransferase 17
194	*BCAT3*	*A0A068U2A4*	Branched-chain-amino-acid aminotransferase 3, chloroplastic
195	*WIN1*	*A0A068TPJ2*	Acetylornithine aminotransferase, chloroplastic/mitochondrial
196	*ARGAH1*	*A0A068TNZ2*	Arginase 1, mitochondrial
197	*ADT5*	*A0A068UH55*	Arogenate dehydratase 5, chloroplastic
198	*CYP79B2*	*A0A068VDX2*	Tryptophan N-monooxygenase 1
199	*ELI5*	*A0A068U6K1*	Tyrosine decarboxylase 1.
200	*TAT*	*A0A068V5Q9*	Tyrosine aminotransferase
201	*SSL11*	*A0A068VL45*	Protein STRICTOSIDINE SYNTHASE-LIKE 11
202	*SSL2*	*A0A068UPH1*	Protein STRICTOSIDINE SYNTHASE-LIKE 2.
203	*OPR2*	*A0A068UR82*	12-oxophytodienoate reductase 2

9. Supplementary Table S10. Generic transcription Pathway

No	**ID *A. thaliana***	**ID *Coffea arabica***	**Associated function**
204	*EMB1674*	*A0A068UQ70*	Protein EMBRYO DEFECTIVE 1674
205	*F17I14.140*	*A0A068TMB2*	Loricrin-like protein.
206	*F22C12.10*	*A0A068UUX5*	WRKY transcription factor.
207	*KNL2*	*A0A068UG78*	Kinetochore-associated protein KNL-2 homolog
208	*NRPA2*	*A0A068UJY6*	DNA-directed RNA polymerase I subunit 2
209	*RPL15*	*A0A068TXH8*	50S ribosomal protein L15, chloroplastic
210	*SKS1*	*A0A068UCG0*	Monocopper oxidase-like protein SKS1
211	*T1O3.7*	*A0A068UZW2*	Putative translation initiation factor eIF-1A
212	*TUFA*	*A0A068VLC2*	Elongation factor Tu, chloroplastic
213	*TUFA-2*	*A0A068U7K4*	Elongation factor Tu, mitochondrial

10. Supplementary Table S11. Glycerolipid Metabolism

No	**ID *A. thaliana***	**ID *Coffea arabica***	**Associated function**
214	*GL2*	*A0A068TP7*	Homeobox-leucine zipper protein GLABRA 2
215	*GPAT1*	*A0A068UDD0*	Glycerol-3-phosphate acyltransferase 1
216	*GPAT2*	*A0A068V426*	Probable glycerol-3-phosphate acyltransferase 2
217	*GPAT3*	*A0A068VLP2*	Probable glycerol-3-phosphate acyltransferase 3
218	*GPAT6*	*A0A068UDZ3*	Glycerol-3-phosphate 2-O-acyltransferase 6
219	*LPAT4*	*A0A068UIW2*	Probable 1-acyl-sn-glycerol-3-phosphate acyltransferase 4

220	*LPAT5*	*A0A068TL75*	Probable 1-acyl-sn-glycerol-3-phosphate acyltransferase 5
221	*LPP2*	*A0A068TXQ8*	Lipid phosphate phosphatase 2
222	*LPP3*	*A0A068U2N3*	Putative lipid phosphate phosphatase 3, chloroplastic
223	*MGD3*	*A0A068UQ78*	Monogalactosyldiacylglycerol synthase 3, chloroplastic
224	*MYB5*	*A0A068UQX2*	Transcription repressor MYB5
225	*RHM1*	*A0A068VDT3*	UDP-4-keto-6-deoxy-D-glucose 3,5-epimerase/UDP-4-keto-L-rhamnose 4-keto-reductase
226	*RHM2*	*A0A068UEJ9*	UDP-4-keto-6-deoxy-D-glucose 3,5-epimerase/UDP-4-keto-L-rhamnose 4-keto-reductase
227	*SBT1.7*	*A0A068U202*	Subtilisin-like protease SBT1.7
228	*SPX2*	*A0A068UPP2*	SPX domain-containing protein 2
229	*SPX3*	*A0A068UML8*	SPX domain-containing protein 3
230	*SQD1*	*A0A068UJ88*	UDP-sulfoquinovose synthase, chloroplastic
